# Bisacurone gel ameliorated burn wounds in experimental rats via its anti-inflammatory, antioxidant, and angiogenic properties

**DOI:** 10.1590/acb382423

**Published:** 2023-08-21

**Authors:** Zengqiang Yan, Shuyan Li, Zhenzhong Gong

**Affiliations:** 1 Inner Mongolia Baogang Hospital – Department of Burns Surgery – Baotou, Inner Mongolia, China.; 2 Inner Mongolia Tongliao Mental Health Center – Department of Cardiology – Tongliao, Inner Mongolia, China.; 3 Fifth Hospital of Harbin City – Department of Burns – Harbin, Heilongjiang Province, China

**Keywords:** Wound Healing, Transforming Growth Factor beta, Vascular Endothelial Growth Factor C

## Abstract

**Purpose:**

To investigate putative mechanism of wound healing for chitosan-based bisacurone gel against secondary burn wounds in rats.

**Methods:**

A second-degree burn wound with an open flame using mixed fuel (2 mL, 20 seconds) was induced in Sprague Dawley rats (male, 180-220 g, *n* = 15, each) followed by topical treatments with either vehicle control (white petroleum gel, 1%), silver sulfadiazine (1%) or bisacurone gel (2.5, 5, or 10%) for 20 days. Wound contraction rate and paw withdrawal threshold were monitored on various days. Oxidative stress (superoxide dismutase, glutathione, malondialdehyde, and nitric oxide), pro-inflammatory cytokines (tumour necrosis factor-alpha, interleukins by enzyme-linked immunosorbent assay), growth factors (transforming growth factor-β, vascular endothelial growth factor C using real time polymerase chain reaction and Western blot assay) levels, and histology of wound skin were assessed at the end.

**Results:**

Bisacurone gel showed 98.72% drug release with a 420.90–442.70 cps viscosity. Bisacurone gel (5 and 10%) significantly (*p* < 0.05) improved wound contraction rate and paw withdrawal threshold. Bisacurone gel attenuated oxidative stress, pro-inflammatory cytokines, and water content. It also enhanced angiogenesis (hydroxyproline and growth factor) and granulation in wound tissue than vehicle control.

**Conclusions:**

These findings suggested that bisacurone gel can be a potential candidate to treat burn wounds via its anti-inflammatory, antioxidant, and angiogenic properties.

## Introduction

Severe burn injuries are physically debilitating and traumatic injuries that affect major organ systems resulting in effective morbidity and mortality. Management of burn pain and rate of wound contraction followed by scar formation are major challenges in wound recovery in a patient with burn injury^1^. Second-degree burns, progressing to deeper tissues with partial thickness, can lead to microvascular dysfunction or death if not treated at an early stage^2^,^3^. These challenges remain the leading cause of long-lasting hospital stays and emotional and economic burdens in these patients^4^. According to the World Health Organization, approximately 120,000 to 180,000 people die every year while seeking burn treatment in low and middle-income countries, posing a serious threat to human life^5^. The annual cost of treating burns in the United States of America alone is one billion dollars, and to address this issue, many researchers have been interested in researching various potential drugs^6^.

Various cellular mechanisms behind microvascular dysfunction have been reported, including upregulation of proapoptotic proteins, inflammatory mediators, and thrombosis due to vascular injury^3^,^7^. In burns, ischemia-reperfusion injury is also one of the main causative factors behind oxidative stress, as it causes the formation of reactive oxygen species (ROS). Thus, the generation of ROS species is responsible for the mechanism behind oxidative stress in burns^8^. Furthermore, reduced angiogenesis and excessive inflammation resulting from burn wounds are the two major factors that play a vital role in delaying the process of skin regeneration and wound healing^9^-^11^. Thus, the involvement of multiple pathophysiological biomarkers during burn injury may assist in managing important challenges, including pain management, and wound contraction rate.

Bisacurone is known for its various pharmacological potential as it inhibits ROS generation and prevents tumor necrosis factor-alpha (TNF-α)-induced nuclear factor kappa-light-chain-enhancer of activated B cells (NF-κB) activation^12^. Bisacurone also exhibits anti-inflammatory properties, thus inhibiting inflammatory proteins such as TNF, inducible nitric oxide (iNOS), and cyclooxygenase-2 (COX-2)^13^. According to a previous study, bisacurone is known to down-regulate the expression of vascular cell adhesion molecules, inhibiting ROS in TNF-α-stimulated human umbilical vein endothelial cells (HUVECs)^14^. Also, it reported that bisacurone down-regulated the expression of NF-κB^15^ by causing the phosphorylation of nuclear factor of kappa light polypeptide gene enhancer in B-cells inhibitor, alpha^16^. The inhibition of these pro-inflammatory cytokines suggests that bisacurone has great anti-inflammatory potential. Thus, the presence of multiple inhibitory potentials of bisacurone might help accelerate the wound healing rate during a burn wound.

The current study was, therefore, conducted to understand the mechanism of action of bisacurone in response to burn wounds in an experimental rat model.

## Methods

### Preparation and characterization of bisacurone gel

Glacial acetic acid (0.5%) was added to half the required water. The weighed amount of chitosan was added and stirred slowly. After the swelling, the remaining amount of water was added and mixed. The gel was kept at room temperature overnight before the application to remove the air bubbles. Then, the required amount of bisacurone solution was added, and the final concentration was 2.5, 5, and 10%. In order to detect the suitability of formulations for topical administration, formulations were evaluated for their characteristic features, such as pH, viscosity, and spreadability.

The three concentrations of bisacurone (i.e., 2.5, 5, and 10%) were selected based on previous studies^17^-^20^.

#### Viscosity

Brookfield viscometer LVDV-E model was used to determine the viscosity of the formulations. The preparations were placed in the sampler tube. The formulations were measured at 50 rpm at 25 ± 0.5 °C.

#### Determination of pH

The pH of the formulations was detected using a calibrated pH meter (Mettler Toledo, Switzerland). Determinations were carried out four times.

#### Spreadability

To determine the spreadability of formulations, 100 mg of blank and bisacurone gel were transferred to the center of a glass plate at 32 °C. This plate was compressed under another plate. After 1 minute, the weight was removed, and the diameter of the spread area (cm) was measured.

### In-vitro drug release studies

An *in-vitro* release study of bisacurone from the chitosan gel formulation was also performed. Briefly, 1 mL of chitosan gel–bisacurone formulation with a concentration of 2 mg/mL was placed in a dialysis sac having a pore size of 12,000 Da, and the sac was immersed in a constantly stirred receiver vessel containing a 15-fold higher volume of the drug-free phosphate buffer (pH 5.8) at 32 ± 0.5 °C. At the designated periods, the sample (3 mL) was removed from the receiver vessel and replenished with fresh buffer. The sample was withdrawn at a predetermined time interval of 0.5, 1, 2, 3, 4, 5, 6, 7, 8, 10, 12, and 24 hours. The samples were then analyzed using a high-performance liquid chromatography on a Shimadzu LC2030 C Prominence-i (Japan) system equipped with a quaternary low-pressure gradient solvent delivery LC2030 pump with high-pressure switching valves, online LC2030 degasser unit, a high-sensitivity LC2030 ultraviolet (UV) detector, and large capacity column oven. Separation was performed in the Kinetex C-18 column (100 A°, 250 mm × 4.6 mm, 5 μm pore size).

The mobile phase consisted of isocratic elution with a low-pressure gradient using 0.2% formic acid:acetonitrile (65:35) with a flow rate of 0.8 mL/min and injection volume of 10 μL. All solutions were degassed and filtered through a 0.45-μm-pore size filter. The column was maintained at 26 °C throughout the analysis, and the ultraviolet (UV) detector was set at 254 nm.

### Determination of the effect of bisacurone gel on burn wound model

#### Animals

Adult Sprague Dawley rats (male, 180-220 g) were obtained from the Fifth Hospital of Harbin City. The experimental protocol (approval number ETH20220715) was approved by the Fifth Hospital of Harbin City and performed by the National Institute of Health Guide for Care and Use of Laboratory Animals guidelines. Rats were housed at 24 ± 1 °C temperature, with a 45–55% relative humidity and 12:12 hours dark/light cycle. The animals had free access to standard pellet chow and filtered water throughout the experimental protocol. All experiments were carried out between 9 a.m. and 5 p.m. in the Department of Burns, The Fifth Hospital of Harbin City, Harbin, Heilongjiang Province, China.

#### Experimental groups, induction of burn wounds, and treatments

Rats were divided into the various groups (*n* = 15, each) as follows:

- Group I: Normal: Rats underwent the shaving procedure without burn injury and were undisturbed for the whole study protocol;
- Group II: Vehicle control: Rats underwent burn injury and were treated with white petroleum gel (1%) topically for 20 days;
- Group III: Silver sulfadiazine (SSD) treated: Rats underwent burn injury and were treated with SSD gel (1%) topically for 20 days;
- Group IV: Bisacurone (2.5%) treated: Rats underwent burn injury and were treated with bisacurone gel (2.5%), topically for 20 days;
- Group V: Bisacurone (5%) treated: Rats underwent burn injury and were treated with bisacurone gel (5%), topically for 20 days;
- Group VI: Bisacurone (10%) treated: Rats underwent burn injury and were treated with bisacurone gel (10%), topically for 20 days.

The second-degree burn was created (except for group I) according to a previously established method^21^. Briefly, the fur was shaved from the dorsal surface of each rat with electric clippers, then rats were anesthetized with 3% pentobarbital (10 mg/kg body weight) by intraperitoneal (i.p.) injection. The rats were positioned in a premade template with a rectangular opening to expose the dorsal skin surface area and protect the remaining skin from direct exposure. The burn area was limited to approximately 25 mm^2^ with the template. A volume of 2 mL of mixed fuel (stock: gasoline, 25 mL; 95% alcohol, 60 mL; rosin, 60 g; glycerol, 5 mL; and xylene, 5 mL) was applied to each wound. The fuel was smeared evenly on the dorsal skin of each rat, lit with an open flame, and allowed to burn for 20 s. The fire was then extinguished as quickly as possible with a wet cloth. Pathological changes in the skin tissue were observed to confirm the injury depth. Bisacurone gel or SSD cream was applied to the wound area starting from the day of the creation.

### Determination of behavioral, biochemical, molecular, and histopathological parameters

#### Measurement of the wound area and wound contraction

The progressive changes in the wound area were recorded with the help of a digital camera (Fuji, S20 Pro, Japan) by an observer blind to the treatment on various days, i.e., 2, 4, 8, 12, 16, and 20. Later, the wound area was measured by tracing the wound on a millimeter scale graph paper. Wound contraction was calculated as a percentage of the original wound area size reduction using a formula reported elsewhere^22^,^23^.

### Determination of the period of epithelization

The epithelialization period was monitored by noticing the days required for the eschar to fall off the burn wound surface without leaving a raw wound behind^24^.

### Determination of paw withdrawal threshold

To assess the painful stimuli during burns, a paw withdrawal threshold to mechano-tactile allodynia (non-noxious mechanical stimuli) was assessed parallelly on various days, i.e., 4, 12, and 20, as previously described in methods^25^,^26^. Briefly, Von-Frey hairs (IITC, Woodland Hills, United States of America) with calibrated bending forces (in g) of different intensities were used to deliver punctuate mechanical stimuli of varying intensities to the rats in each group. The criterion for the threshold value, in grams, was equal to the filament evoking a withdrawal of the paw five times out of 10 trials, i.e., 50% response.

### Determination of blood cell count

On day 21, rats were anesthetized under ethereal anesthesia, and a retro-orbital puncture technique was used to collect blood in a tube containing EDTA-Na salt. White blood cells (WBC), platelet, lymphocyte, neutrophil, monocyte, eosinophil, and basophil were measured.

### Determination of endogenous antioxidant enzymes levels, water, and hydroxyproline content

Post-blood collection, rats were sacrificed by cervical dislocation, and wound tissues were rapidly removed and stored at -80 °C for biochemical parameters. A portion of would tissue from random animals (n = 6) was subjected to determine superoxide dismutase (SOD), reduced glutathione (GSH), MDA, and nitric oxide (NO), which was determined in wound tissue as described previously^26^,^27^. Another portion of would tissue (n = 6) was used to assess water and hydroxyproline content as described previously^28^,^29^.

### Determination of TNF-α, IL-1β, and IL-6

TNF-α, interleukin (IL)-1β, and IL-6 levels were determined in the wound tissue (in a portion of would tissue from another set of random animals [n = 6]) using rat enzyme-linked immunosorbent assay (ELISA) quantitation kit (Bethyl Laboratories Inc., Montgomery, TX, United States of America).

### Determination of TGF-β and VEGF-c mRNA and protein expressions

The remaining portion of would tissue from another set of random animals (n = 6) was used to determine levels of transforming growth factor beta (TGF-β) and vascular endothelial growth (VEGF)-c mRNA expressions using a reverse transcription (RT)-polymerase chain reaction (PCR) approach as described previously^26^ whereas protein expressions were assessed by Western blot assay using previously described method^30^.

During RT-PCR, total RNA was extracted from skin tissues according to the manufacturer’s instructions (MP Biomedicals India Private Limited, India). The PCR mixture was amplified in a DNA thermal cycler (Eppendorf India Ltd, Chennai, India) using gene-specific primers. The primer for TGF-β (up-stream: 5’-CCCTGATGAGATCGAGTACATCTT-3’; down-stream: 5’-ACCGCCTCGGCTTGTCAC-3’; amplicon size: 165), VEGF-c (up-stream: 5’-GTTCTTCAATACGTCAGACATTCG-3’; down-stream: 5’-CATTATCTTTGCTGTCACAAGAGC-3’; amplicon size: 309) and β-actin (up-stream: 5’-GTCACCCACACTGTGCCCATCT-3’; down-stream: 5’-ACAGAGTACTTGCGCTCAGGAG-3’; amplicon size: 764) was provided by the manufacturer (MP Biomedicals India Private Limited, India). PCR products were run on 1% agarose gel and stained with ethidium bromide. Gene expression was assessed by generating densitometry data for band intensities in different sets of experiments by analyzing the gel images on the Image J program (Version 1.33, Wayne Rasband, National Institutes of Health, Bethesda, MD, United States of America). The band intensities were compared with constitutively expressed β-actin, which controlled sample loading and integrity. The intensity of mRNAs was standardized against that of the β-actin mRNA from each sample, and the results were expressed as PCR-product/β-actin mRNA ratio..

The protein levels of TGF-β and VEGF-c in wound tissue were estimated using Western blot assay. Briefly, wound tissue sonicated in tissue protein extraction reagent (Thermo Fisher Scientific, Inc.). The lysates were centrifuged at 10,000 xg for 10 min at 4 °C. Protein concentration was determined using a bicinchoninic acid (BCA) assay kit (Beyotime Shanghai, China) on ice for 30 minutes. Equal amounts of extracted protein samples (50 μg) were separated by 10% sodium dodecyl sulfate-polyacrylamide gel electrophoresis (SDS-PAGE) and transferred onto polyvinylidene difluoride membranes. The membranes were blocked with 5% non-fat dry milk at 37 °C for 1 hour and incubated overnight at 4 °C with the primary antibodies that recognized TGF-β, VEGF-c, and GAPDH. Anti-rabbit horseradish-linked IgG was used as the secondary antibody, which was incubated at 37 °C for 2 hours. Protein bands were visualized using the chemiluminescent kit (Bio-Rad Laboratories, Inc.), and GAPDH served as the loading control.

### Histopathological examination

A specimen sample of wound tissue (n = 3) was used to evaluate the histopathological alterations. Samples were fixed in 10% buffered formalin, processed, and blocked with paraffin, then sectioned into 5 μm and stained with hematoxylin and eosin (H and E). Photomicrographs were captured at a magnification of 100 X. Re-epithelization in the epidermis, mononuclear and polymorphonuclear cells, neovascularization, or new vessel formation were analyzed to score the epidermal or dermal remodeling.

### Sample size and statistical analysis

The sample size was calculated based on the power analysis method considering 30% expected attrition using Eq. 1:


$$\text{Corrected sample size}=\text{Sample size}/(1-[\%\text{attrition/100}]{)}^{31}$$ (1)

Data analysis was conducted using GraphPad Prism 5.0 software (GraphPad, San Diego, CA, United States of America) and reported as mean ± standard error mean (SEM) with *P* < 0.05 considered statistically effective. A post hoc analysis was conducted using Tukey’s multiple ranges (for parametric tests including behavioral, biochemical, and molecular parameters [except epithelization period]) and the Kruskal-Wallis followed by Mann-Whitney’s multiple comparison tests (non-parametric tests including for epithelization period and histopathological analysis of skin tissue) during one-way analysis of variance (ANOVA). Data were compared between normal, vehicle control, and treatment group and within the treatment group.

## Results

### Characterization of bisacurone gel

**Figure 1:**
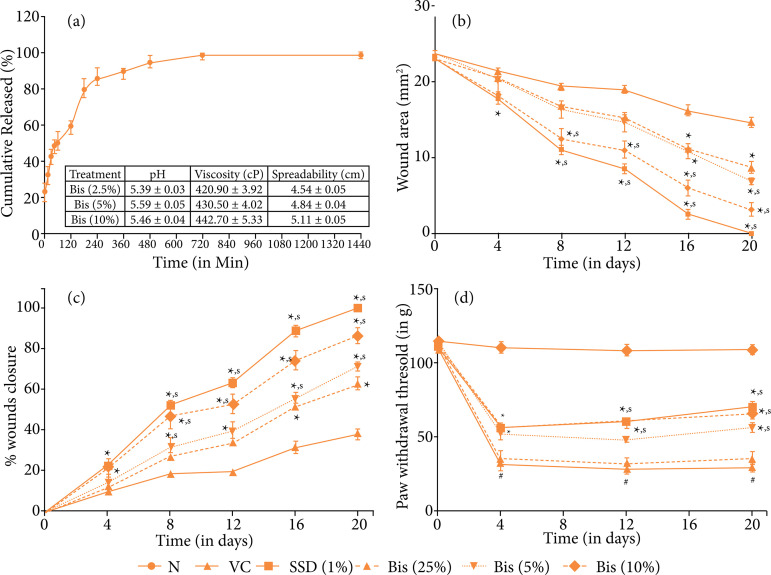
**(a)** *In-vitro* releases profile of bisacurone from chitosan-gel and formulation characterization, **(b)** the effect of bisacurone treatment on wound area, **(c)** percent wound closure, and **(d)** paw withdrawal threshold in rats^@^.

^@^Data are expressed as mean ± standard error mean (n = 6). Data of wound area, percent wound closure, and paw withdrawal threshold are analyzed by two-way analysis of variance (ANOVA) followed by Tukey’s multiple range test for each parameter separately; ^#^ *p < 0.05* as compared to normal, *p < 0.05 as compared to vehicle control; ^$^ *p < 0.05* as compared to one another; VC: vehicle control; SSD (1%): silver sulfadiazine (1%) treated group; Bis (2.5%): bisacurone (2.5 %) treated group; Bis (5%): bisacurone (5%) treated group; Bis (10%): bisacurone (10%) treated group. Source: Authors.

The release of bisacurone from the chitosan gel was found to be 98.72 ± 0.59% for 24 hours. The release kinetics from the gel formulation was found to be the first order. This result indicated that the release rate of bisacurone from the gel varies with time. The pH of the bisacurone gel (2.5, 5, and 10%) was 5.39 ± 0.03, 5.59 ± 0.05, and 5.46 ± 0.04, respectively, which lie in the normal pH range of human skin, whereas the viscosity was 420.90 ± 3.92, 430.50 ± 4.02, and 442.70 ± 5.33 cps, respectively, which indicated that as the torque and shear stress increases. The spreadability time of these formulations was 4.54 ± 0.05, 4.84 ± 0.04, and 5.11 ± 0.05 cm, respectively, indicating they are nearly the same in terms of applicability or spreading capacity (Fig. 1a).

### Effect of bisacurone treatment on the rate of wound contraction in rats

When compared to vehicle control on day 4, the SSD (1%) treated group began to exhibit a significantly higher (*p* < 0.05) percentage of wound closure. In comparison to vehicle control on day 8, bisacurone (10%) demonstrated a remarkably greater (*p* < 0.05) percentage of wound closure. This pattern continued for the bisacurone (10%) and SSD (1%) treated groups on days 12, 16, and 20. It was on day 16 when bisacurone (5%) demonstrated a more significant (*p* < 0.05) improvement than the vehicle control group. On day 20, bisacurone gel (5% and 10%) showed a significant (*p* < 0.05) enhancement in wound closure and better-wound healing than vehicle control (Figs. 1b and 1c).

### Effect of bisacurone treatment on an epithelization period in rats

When compared to a vehicle control group, the epithelization time was significantly (*p* < 0.05) shorter for the SSD (1%). Likewise, for bisacurone (5 and 10%) groups, the epithelization time was significantly shorter (*p* < 0.05) when compared with vehicle control, while the shortening time was non-significant for the bisacurone (2.5%) (Table 1).

**Table 1:** Effect of bisacurone treatment on epithelization period, water content, hydroxyproline level, and protein levels in wound tissue of rats@.

Treatment	Epithelization period (days)	Water content (%)	Hydroxyproline ( μg/mg tissue)	Protein ( μg / mg tissue)
Normal	-	35.73 ± 2.42	107.70 ± 4.56	35.50 ± 0.68
VC	18.33 ± 0.33	54.85 ± 1.97#	45.04 ± 3.67#	53.66 ± 1.28#
SSD (1%)	8.17 ± 0.40*,$	36.84 ± 2.31*,$	94.54 ± 4.20*,$	40.45 ± 1.25*,$
Bis (2.5%)	16.67 ± 0.42	50.81 ± 2.55	55.75 ± 3.54	51.76 ± 0.87
Bis (5%)	14.33 ± 0.33*,$	47.79 ± 1.99*,$	81.25 ± 3.65*,$	46.69 ± 0.48*,$
Bis (10%)	10.50 ± 0.50*,$	41.99 ± 1.88*,$	93.63 ± 3.69*,$	42.44 ± 0.94*,$

@ Data are expressed as mean ± standard error mean (n = 6) and analyzed by Kruskal-Wallis followed by Mann-Whitney’s multiple comparison tests for the epithelization period, whereas one-way analysis of variance (ANOVA) followed by Tukey’s multiple range test for water content, hydroxyproline level, and protein levels;

# *p < 0.05* as compared to normal;

* *p* < 0.05 as compared to vehicle control;

$ *p* < 0.05 as compared to one another;

VC: vehicle control; SSD (1%): silver sulfadiazine (1%) treated group; Bis (2.5%): bisacurone (2.5 %) treated group; Bis (5%): bisacurone (5%) treated group; Bis (10%): bisacurone (10%) treated group. Source: Authors.

### Effect of bisacurone treatment on paw withdrawal threshold in rats

On days 4, 12, and 20, vehicle control showed a significant (*p* < 0.05) response with a lower paw withdrawal threshold than the normal group. Day 4 indicated a considerably (*p* < 0.05) higher response in paw withdrawal threshold in the bisacurone (10%) and SSD (1%) treated groups than in vehicle control group. On day 12, a significant increase (*p* < 0.05) in paw withdrawal threshold was produced in SSD (1%) and bisacurone (5 and 10%) treated groups in comparison with vehicle control. On day 20, the groups treated with bisacurone (5% and 10%) demonstrated a significantly (*p* < 0.05) enhanced paw withdrawal threshold than a vehicle control group, while over the period of 20 days bisacurone (2.5%) did not significantly alter the responsiveness to the paw withdrawal threshold (Fig. 1d).

### Effect of bisacurone treatment on blood cell count

WBC, platelet, neutrophils, and monocyte counts were considerably (p < 0.05) higher in vehicle control rats than in normal rats. Compared to vehicle control rats, rats treated with SSD (1%) had significantly (*p* < 0.05) reduced levels of WBC, platelet, neutrophils, and monocytes. WBC, platelet, neutrophils, and monocyte levels were significantly (*p* < 0.05) suppressed in the bisacurone (5 and 10%) rats than in vehicle control rats (Table 2).

**Table 2:** Effect of bisacurone treatment on the white blood cell, platelet, neutrophil, and monocyte count in rats@.

Treatment	WBC count (X10^3^/mL)	Platelet count (X10^9^/mL)	Neutrophils (%)	Monocytes (%)
Normal	1.33 ± 0.21	407.20 ± 5.22	9.17 ± 0.65	1.33 ± 0.21
VC	4.00 ± 0.26#	500.80 ± 6.00#	16.00 ± 0.93#	4.00 ± 0.26#
SSD (1%)	2.00 ± 0.26*,$	412.50 ± 2.53*,$	11.50 ± 0.43*,$	1.83 ± 0.17*,$
Bis (2.5%)	4.00 ± 0.26	495.30 ± 6.92	14.83 ± 0.79	3.67 ± 0.33
Bis (5%)	3.00 ± 0.26*,$	465.20 ± 4.74*,$	13.00 ± 0.63*,$	2.83 ± 0.40*,$
Bis (10%)	2.00 ± 0.26*,$	440.50 ± 6.84*,$	10.33 ± 0.56*,$	2.50 ± 0.22*,$

@ Data are expressed as mean ± standard error mena (n = 6) and analyzed by one-way analysis of variance (ANOVA) followed by Tukey’s multiple range test for each parameter separately;

# *p* < 0.05 as compared to normal;

* *p* < 0.05 as compared to vehicle control;

$ *p* < 0.05 as compared to one another;

VC: vehicle control; SSD (1%): silver sulfadiazine (1%) treated group; Bis (2.5%): bisacurone (2.5%) treated group; Bis (5%): bisacurone (5%) treated group; Bis (10%): bisacurone (10%) treated group; WBC, white blood cell. Source: Authors.

### Effect of bisacurone treatment on the oxido-nitrosative stress in wound tissue of rats

GSH and SOD levels were considerably (*p* < 0.05) lower in vehicle control group than in the normal group. In comparison to a vehicle control group, the SSD (1%) treatment group had significantly elevated (*p* < 0.05) levels of GSH and SOD. Bisacurone (5 and 10%) groups showed considerably greater (*p* < 0.05) GSH and SOD levels than vehicle control group, but bisacurone (2.5%) did not have any significant effects on this (Table 3).

**Table 3:** Effect of bisacurone treatment on the oxido-nitrosative stress and levels of TNF-α, IL-1β, and IL-6 in wound tissue of rats@.

Treatment	SOD (U /mg of protein)	GSH ( μg/mg of protein)	MDA (nM/ mg of protein)	NO( μg/mL)	TNF-α(pg/mL)	IL-1β(pg/mL)	IL-6(pg/mL)
Normal	5.82 ± 0.22	10.44 ± 0.34	5.22 ± 0.67	202.50 ± 15.31	14.69 ± 0.48	4.91 ± 0.36	12.81 ± 2.17
VC	2.46 ± 0.17#	3.46 ± 0.65#	28.70 ± 0.73#	830.30 ± 22.29#	44.29 ± 0.69#	10.10 ± 0.32#	63.14 ± 3.05#
SSD (1%)	5.39 ± 0.20*,$	10.14 ± 0.88*,$	12.14 ± 0.62*,$	447.40 ± 18.41*,$	23.55 ± 0.72*,$	6.74 ± 0.43*,$	19.50 ± 2.55*,$
Bis (2.5%)	2.42 ± 0.27	4.31 ± 0.50	26.08 ± 0.90	755.50 ± 18.94	42.56 ± 0.77	9.09 ± 0.41	61.69 ± 2.38
Bis (5%)	3.76 ± 0.20*,$	6.44 ± 0.79*,$	20.23 ± 0.59*,$	542.30 ± 17.77*,$	33.16 ± 0.82*,$	8.10 ± 0.38*,$	42.72 ± 2.96*,$
Bis (10%)	4.49 ± 0.17*,$	8.60 ± 0.55*,$	13.98 ± 0.71*,$	450.70 ± 18.81*,$	26.47 ± 0.70*,$	6.76 ± 0.49*,$	32.50 ± 2.87*,$

@ Data are expressed as mean ± standard error mean (n = 6) and analyzed by one-way analysis of variance (ANOVA) followed by Tukey’s multiple range test for each parameter separately;

# *p* < 0.05 as compared to normal;

* *p* < 0.05 as compared to vehicle control;

$ *p* < 0.05 as compared to one another;

VC: vehicle control; SSD (1%): silver sulfadiazine (1%) treated group; Bis (2.5%): bisacurone (2.5 %) treated group; Bis (5%): bisacurone (5%) treated group; Bis (10%): bisacurone (10%) treated group; SOD: superoxide dismutase; GSH: reduced glutathione; MDA: lipid peroxidation; NO: nitric oxide; TNF-α: tumor necrosis factor-alpha; IL: interleukin. Source: Authors.

Compared to the normal group, the levels of MDA and NO in vehicle control were considerably (*p* < 0.05) higher. MDA and NO activity were significantly reduced (*p* < 0.05) in the SSD (1%) treated group than in vehicle control group. The levels of MDA and NO were significantly lower (*p* < 0.05) in the bisacurone (5 and 10%) groups than vehicle control. The bisacurone (10%) group showed the lowest MDA and NO activity levels among the three treated groups. However, MDA and NO activity level was lower in the SSD (1%) treated group than bisacurone. The bisacurone (2.5%) treatment did not reach statistical significance or demonstrate a major improvement in NO and MDA activities (Table 3).

### Effect of bisacurone treatment on water content and hydroxyproline level in wound tissue of rats

Compared to the normal group, the amount of hydroxyproline in the vehicle control group was considerably (*p* < 0.05) lower. The SSD (1%) treated group showed a significantly (*p* < 0.05) greater level of hydroxyproline than vehicle control. The bisacurone (5 and 10%) treated groups had considerably (*p* < 0.05) higher levels of hydroxyproline than a vehicle control group, but the hydroxyproline level in the bisacurone (2.5%) was statistically non-significant (Table 1).

Compared to the normal group, the percentage of water content in vehicle control group was considerably higher (*p* < 0.05). On the other hand, the group that received SSD (1%) treatment had significantly decreased (*p* < 0.05) water content than vehicle control group. Furthermore, compared to vehicle control, the proportion of water content in the bisacurone (5 and 10%) treated groups was considerably lower (*p* < 0.05), whereas bisacurone (2.5%) had no significant effect in reducing the water content in the wounded tissues of rats (Table 1).

### Effect of bisacurone treatment on TNF-α, IL-1β, and IL-6 levels in wound tissue of rats

TNF-α, IL-1β, and IL-6 levels were considerably (*p* < 0.05) higher in the vehicle control group than in the normal group. Compared to vehicle control group, the SSD (1%) treated group had reduced levels of TNF-α, IL-1β, and IL-6 significantly (*p* < 0.05). TNF-α, IL-1β, and IL-6 concentrations were significantly (*p* < 0.05) suppressed in the bisacurone (5 and 10%) groups than vehicle control group (*p* < 0.05), but not in the (2.5%) bisacurone group (Table 3).

### Effect of bisacurone treatment on TGF-β and VEGF-c mRNA expressions in wound tissue of rats

**Figure 2:**
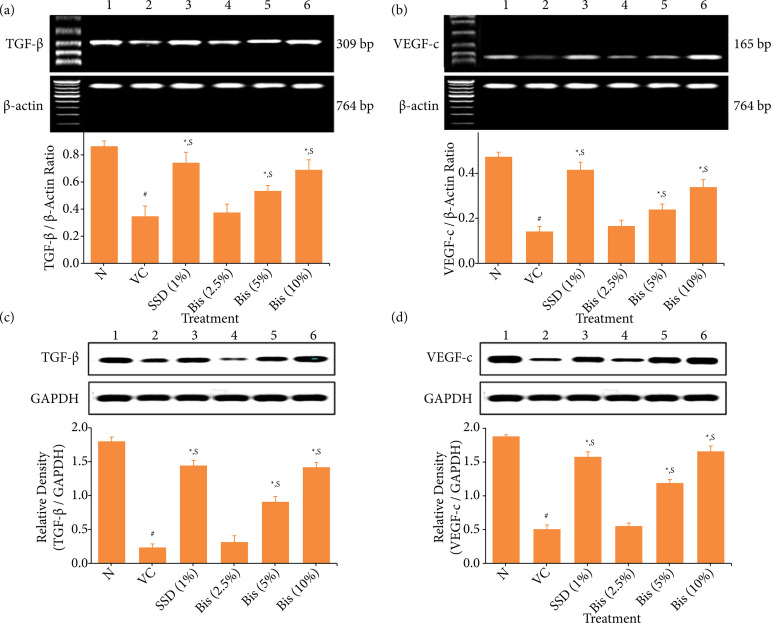
Effect of bisacurone treatment on mRNA expressions of **(a)** TGF-β and **(b)** VEGF-c, and **(c)** protein expressions of TGF-β, and **(d)** VEGF-c in wound tissue of rats^@^.

^@^Data are expressed as mean ± standard error mean (n = 6) and analyzed by one-way analysis of variance (ANOVA) followed by Tukey’s multiple range test for each parameter separately; ^#^ *p* < 0.05 as compared to normal; *p < 0.05 as compared to vehicle control; ^$^ *p < 0.05* as compared to one another; VC: vehicle control; SSD (1%): silver sulfadiazine (1%) treated group; Bis (2.5%): bisacurone (2.5 %) treated group; Bis (5%): bisacurone (5%) treated group; Bis (10%): bisacurone (10%) treated group; TGF-β, Transforming growth factor-β; VEGF, vascular endothelial growth factor; GAPDH: glyceraldehyde-3-phosphate dehydrogenase. Source: Authors.

The mRNA expression levels of the VEGF-c and TGF-β in the skin tissues of vehicle control group showed a greater significant downregulation (*p* < 0.05) than normal group. The SSD (1%) treated group showed a significantly higher (*p* < 0.05) level of VEGF-c and TGF-β than vehicle control. When compared to vehicle control group, the bisacurone 5 and 10% groups showed a significant upregulation (*p* < 0.05) of VEGF-c and TGF-β mRNA expression, but this level was lower as compared to SSD (1%) treated group (*p* < 0.05), while the results were not significant for the bisacurone (2.5%) (Figs. 2a and 2b).

### Effect of bisacurone treatment on TGF-β and VEGF-c protein expressions in wound tissue of rats

The relative expressions of TGF-β and VEGF-c protein were considerably lower (p < 0.05) in the vehicle control group than in the normal group, while the relative expressions of VEGF-c and TGF-β proteins were strikingly enhanced (*p* < 0.05) in SSD (1%) treated group than vehicle control. Bisacurone (5 and 10%) also showed significantly higher (*p* < 0.05) expression of TGF-β and VEGF-c protein as compared to vehicle control, which showed a positive impact on the wound healing process and inhibitory effect of scar formation by angiogenic protein VEGF-c and fibrosis-related protein TGF-β. Compared to the standard SSD (1%) treated group, the VEGF-c protein expression in the bisacurone (10%) treated group was higher. The results for the bisacurone (2.5%) group were statistically non-significant and did not demonstrate any protective effects against scarring and the wound healing process (Figs. 2c and 2d).

### Effect of bisacurone treatment on histopathological alterations in skin tissue of rats

**Figure 3:**
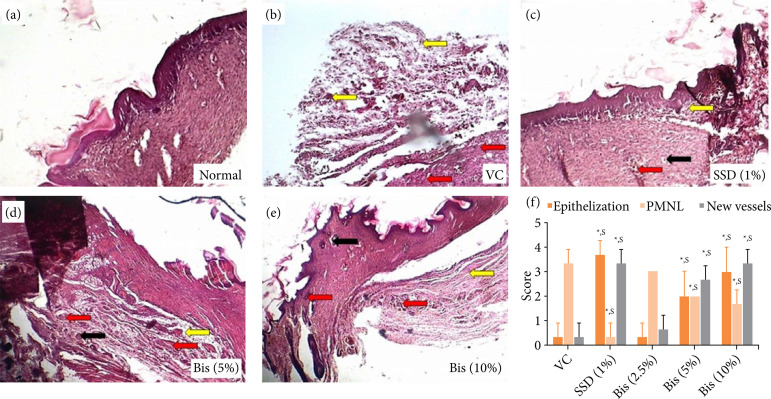
Effect of bisacurone treatment on wound histology in rats. **(a)** Photomicrographs of sections of wound skin from normal, **(b)** vehicle control, **(c)** silver sulfadiazine (1%), **(d)** bisacurone (5%), and **(e)** bisacurone (10%) treated group. **(f)** The quantitative analysis of the effect of bisacurone treatment on wound histology^@^.

^@^Data are expressed as mean ± standard error mean (n = 6) and analyzed by Kruskal-Wallis, followed by Mann-Whitney’s multiple comparison tests for each parameter separately; ^#^ *p* < 0.05 as compared to normal; *p < 0.05 as compared to vehicle control; ^$^ *p* < 0.05 as compared to one another. Hematoxylin & eosin-stained staining at 100 X; yellow arrow: epithelization; black arrow: new vessel formation; red arrow: polymorphonuclear neutrophil leukocytes infiltration. Source: Authors.

Figure 3a depicts the normal architecture of skin tissue without any edema, inflammatory infiltration, or necrosis. In the vehicle control group, the untreated wounds showed delayed healing activity where there was less re-epithelization with very few epithelial epidermal layers. The dermis layer showed high infiltration of macrophages, leukocytes, and neutrophils with no formation of new blood vessels and no granulation (Fig. 3b). The healed wounds in the bisacurone (5 and 10%) treated groups were mostly re-epithelized with many layers of epidermal epithelial cells present in them than vehicle control group (*p* < 0.05). The dermis layer was lightly infiltrated with neutrophils and macrophages, whereas necrotic tissue was healed and replaced by granulation tissue rich in fibroblasts with newly formed blood vessels (Figs. 3d and 3e). The tissue sections of the SSD (1%) treated group showed complete re-epithelization with significantly (*p* < 0.05) increased granulation and minimal infiltration of macrophages, neutrophils, and lymphocytes with newly formed blood vessels (Fig. 3c) than vehicle control group (Fig. 3f).

## Discussion

The present study observed positive results with bisacurone gel in response to inflammation and oxidative stress on burn wounds in rats. The modulation of inflammatory response in the present study was observed by estimating various inflammatory markers like TNF-α, IL-1β, and IL-6 levels involved in the inflammation process^32^,^33^. Compared to vehicle control, bisacurone gel showed a greater response in lowering the levels of these pro-inflammatory mediators and reducing the risk of multiplication of other chemotactic signal molecules at the wound site, promoting wound healing.

Studies have shown that, with the increased rate of epithelization, wound healing is promoted due to the restoration of the epidermis by keratinocytes. A wound is considered to be healed only in the presence of re-epithelization^34^. In the present study, bisacurone gel significantly shortened the epithelization period compared to vehicle control due to re-epithelization and migration of keratinocytes from the wound edge to the denuded area. Hydroxyproline is an important biomarker for collagen synthesis and acts as an indicator of collagen level, which provides stability and structural growth to the skin and organs. The breakdown of collagen during wounds releases free hydroxyproline, thus making it a marker for collagen content in the tissues^35^. Increased hydroxyproline levels indicate collagen synthesis and cellular proliferation, which aids in wound healing. In our study, there was a significant increase in hydroxyproline levels with bisacurone gel that shows collagen stability during the wound healing process than in the control group.

The wound healing cascade is majorly impacted by inflammation and oxidative stress, and increased level of ROS results in the alteration of GSH, SOD, MDA, and NO^32^,^36^,^37^. Superoxide dismutase and GSH are the antioxidant enzymes that defend against ROS in burns. It has been previously studied that SOD levels decrease after burns in tissue plasma due to increased activity of SOD against oxidative stress^38^-^40^. Exogenous SOD therapy has been found to be clinically effective against lipid peroxidation in burn patients^41^. However, tissue ischemia and burn-induced oxidative stress cause the depletion of intracellular GSH levels^42^-^44^. Previous studies indicate that drugs with antioxidant capacity can stimulate GSH activity during burns^45^. Nitric oxide is a free radical produced by the NO synthase enzyme^46^,^47^. When NO reacts with superoxide anion, it generates oxynitrite or peroxynitrite, creating a high level of oxidative stress in tissues to imbalance the oxygen-oxidation reaction^48^,^49^. A previous study has indicated that inhibiting the activity of NO through anti-inflammatory action can reduce oxidative stress in burns^50^. In the current study, bisacurone gel exerted its antioxidant potential, thus elevating the depleted levels of antioxidant enzymes (such as SOD and GSH), reducing MDA and NO levels, and up-regulating stages of wound healing.

Angiogenesis, which creates new blood vessels from old ones by entering the wound clot and assembling into a microvascular network throughout the granulation tissue, is essential for the healing of wounds. Angiogenic cytokines engaged in wound angiogenesis include VEGF and TGF-β, which act as angiogenic stimulators and stimulate endothelial cell proliferation^51^,^52^. VEGF also regulates integrin receptors for forming new blood vessels^53^,^54^. Transforming growth factor-beta act as a chemoattractant molecule for fibroblasts, neutrophils, and macrophages and carry out endothelial cell proliferation and deposition of extracellular matrix on wound site^55^,^56^. The histopathology states that, in the present study, the newly formed blood vessels in the control group were very less due to decreased angiogenesis, endothelial cell growth, and migration. Decreased expression of VEGF and TGF-β due to tissue hypoxia in burns is the major reason behind no new blood vessel formation and reduced epithelization. Bisacurone gel showed increased angiogenesis, endothelial cell migration, proliferation, and high expression of VEGF and TGF, indicating accelerated wound healing.

It is well known that the production of VEGF by endothelial and smooth muscle cells in response to inflammation aids in wound healing promotes angiogenesis, deposition of collagen synthesis, and endothelial cell formation^34^,^57^,^58^. VEGF stimulates endothelial cells and is involved in endothelial cell proliferation and migration at the target site. It binds to KDR receptors and stimulates the activity of NO synthase, which enhances the stimulation of NO and increases vasodilation and vascular permeability at the wound site^59^,^60^. After debridement, platelets release VEGF into the wound, encouraging macrophage-mediated migration and proliferation of endothelial cells. It has been shown that VEGF stimulates collagen synthesis and keratinocyte migration through fibroblasts. Also, VEGF secretion triggers the release of other growth factors, which promote repair^61^-^63^. In our study, VEGF levels were remarkably elevated with bisacurone gel compared with vehicle control, showing accelerated wound healing, vascular permeability, endothelial cell migration, and new blood vessel formation at the injury site. Increased expressions of TGF-β with bisacurone gel showed that it facilitates fibroblast migration and proliferation and it is implicated in modulating collagen synthesis during wound healing.

The exact mechanism behind pain during burns is elusive, but there is a direct relationship between stress/anxiety and pain. During painful stimuli in burns, C fibers release substances P and neurokinin A that stimulate the release of pro-inflammatory cytokines, which causes inflammatory pain signals in response to the stress^64^-^66^. This stress response can generate pain at neuroendocrine, nociceptive, and inflammatory levels^67^,^68^. In addition, the stress signals can increase glucocorticoid release, reducing cellular differentiation and proliferation^69^,^70^. In the current study, during the wound healing process with bisacurone gel, nociceptive pain levels declined substantially compared to vehicle control. The evaluation of characteristics such as increased pain threshold and delayed withdrawal reaction to pain made it clear that bisacurone gel benefits pain management during burns.

Recently, herbal ointments like *Albizia julibrissin* gel and *Aloe vera* gel showed improved healing without adverse effects in burn patients^71^, while a topical application of synthetic drugs (such as SSD, cerium nitrate, and silver nitrate treatments) for the treatment of burn wounds, however, can result in adverse effects such as crystalluria or methemoglobinemia, renal disease (following nitrofurazone treatments), ototoxicity (following chlorhexidine treatments), and thyroid and renal dysfunction (after povidone-iodine treatment)^72^. Moreover, topical formulations like bisacurone with antioxidant, anti-inflammatory, and antimicrobial action should be investigated more in treating burns as they might show fewer adverse effects on systemic functions when tested clinically.

## Conclusion

Bisacurone improved the process of healing in secondary burn wounds in experimental rats. Bisacurone exhibited its potential via inhibition of inflammatory cytokines (tumor necrosis factor-alpha, interleukins) and oxidative stress (SOD, GSH, and MDA), thus accelerating the process of angiogenesis (hydroxyproline, TGF-β, and VEGF-c), fibroblast proliferation, and endothelial cell migration. These findings suggest that bisacurone gel can be a potential candidate to treat secondary wounds with its anti-inflammatory, antioxidant, and angiogenic properties.

## Data Availability

The data will be available upon request.
